# Supplementary material for: Electrical Conductivities of Narrow-Bandgap Polymers with Two Types of π-Conjugated Post-Crosslinking
Source: Polymers (Basel). 2022 Jun 17;14(12):2472. doi: 10.3390/polym14122472 (PMC9229259; doi:10.3390/polym14122472)
Supplement: Supplementary file 1 [file polymers-14-02472-s001.zip › polymers-1743401-supplementary.pdf]

polymers

MDPI

Supplementary Materials

Electrical Conductivities of Narrow-Bandgap Polymers with Two Types of π-Conjugated Post-Crosslinking

Hao-xuan Guo *, Hiroshi Takahara, Yusuke Imai and Hiroyuki Aota *

Department of Chemistry and Materials Engineering, Kansai University, Suita, Osaka 564-8680, Japan; takaharahiroshi895@gmail.com (H.T.); imaiyusuke.425752@gmail.com (Y.I.)

* Correspondence: hx-guo@kansai-u.ac.jp (H.-x.G.); aota@kansai-u.ac.jp (H.A.); Tel.: +81-06-6368-0831

(1) ^{1}H-NMR spectra of the non-conjugated polymers

P1: **Figure S1** shows the chemical structure and ^{1}H-NMR spectrum of **P1**. The peaks at 9–10, 6.5, and 5.5 ppm are assigned to Pyr protons (a, c, and b). The peaks at 7.08 and 7.73 ppm are assigned to the BS protons (d and e).

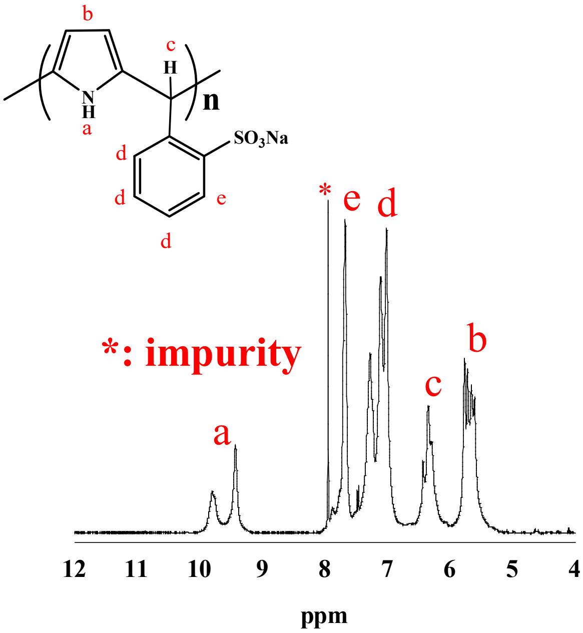

Figure S1. ^{1}H-NMR spectrum of **P1** in DMSO-d$_6$.

(2) ^{1}H-NMR spectrum of the narrow-bandgap polymer

Figure S2 shows the chemical structure and ^{1}H-NMR spectrum of the π-conjugated polymer **Pyr(non)**. A spectral broadening from 6 to 8.2 ppm is observed for **Pyr(non)**. There are no characteristic N-H peaks at ~9.5 ppm in the ^{1}H-NMR spectrum, owing to π-conjugation in the polymer.

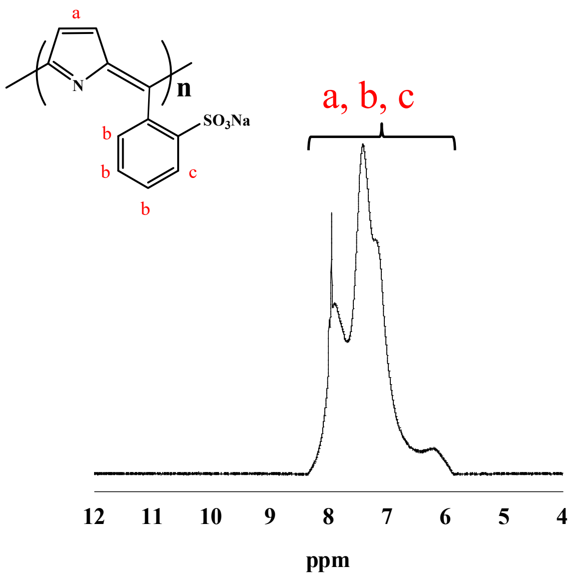

Figure S2. ^{1}H-NMR spectrum of **Pyr(non)** in DMSO-d$_6$.

(3) Non-conjugated Polymer having pyrene

The non-conjugated polymer with pyrene was prepared as follows: a solution of *p*-TS (0.016 g, 0.085 mmol) dissolved in DMF (1.0 mL) was added to a solution of Pyr (0.168 g, 2.5 mmol) and BS (0.521 g, 2.5 mmol) dissolved in DMF (3.0 mL) at 10 °C. The mixed solution was stored in the dark at 10 °C for 24 h, after which a solution containing 1-pyrenecarboxaldehyde (0.058 g, 0.25 mmol) and Pyr (0.017 g, 0.25 mmol) dissolved in DMF (2.0 mL) was added to the reaction mixture and subsequently stored in the dark for 6 h. The reaction was quenched by the addition of sodium carbonate aqueous solution (5 wt%, 3.2 mL). Isopropyl alcohol (80 mL) was added to the reaction mixture. The resulting precipitate, a non-conjugated polymer, was purified by serial reprecipitations, twice from DMF/isopropyl alcohol (8 mL/80 mL) and twice from water/isopropyl alcohol (6 mL/80 mL), and was subsequently dissolved in water. The polymer was obtained by freeze-drying **Pyr-BS(pyrene) (**0.495 g).

Figure S3a shows the absorption spectra of 1-pyrenecarboxaldehyde and pyrene. Figure S3b shows the absorption spectra before and after the reaction of **P1** and 1-pyrenecarboxaldehyde. The absorption profiles of 1-pyrenecarboxaldehyde disappear after the reaction of **P1** with 1-pyrenecarboxaldehyde, whereas those of pyrene appear. Therefore, the reaction of **P1** with aldehydes is demonstrated.

Figure S3c shows the absorption spectrum of the polymer containing pyrene (after purification).

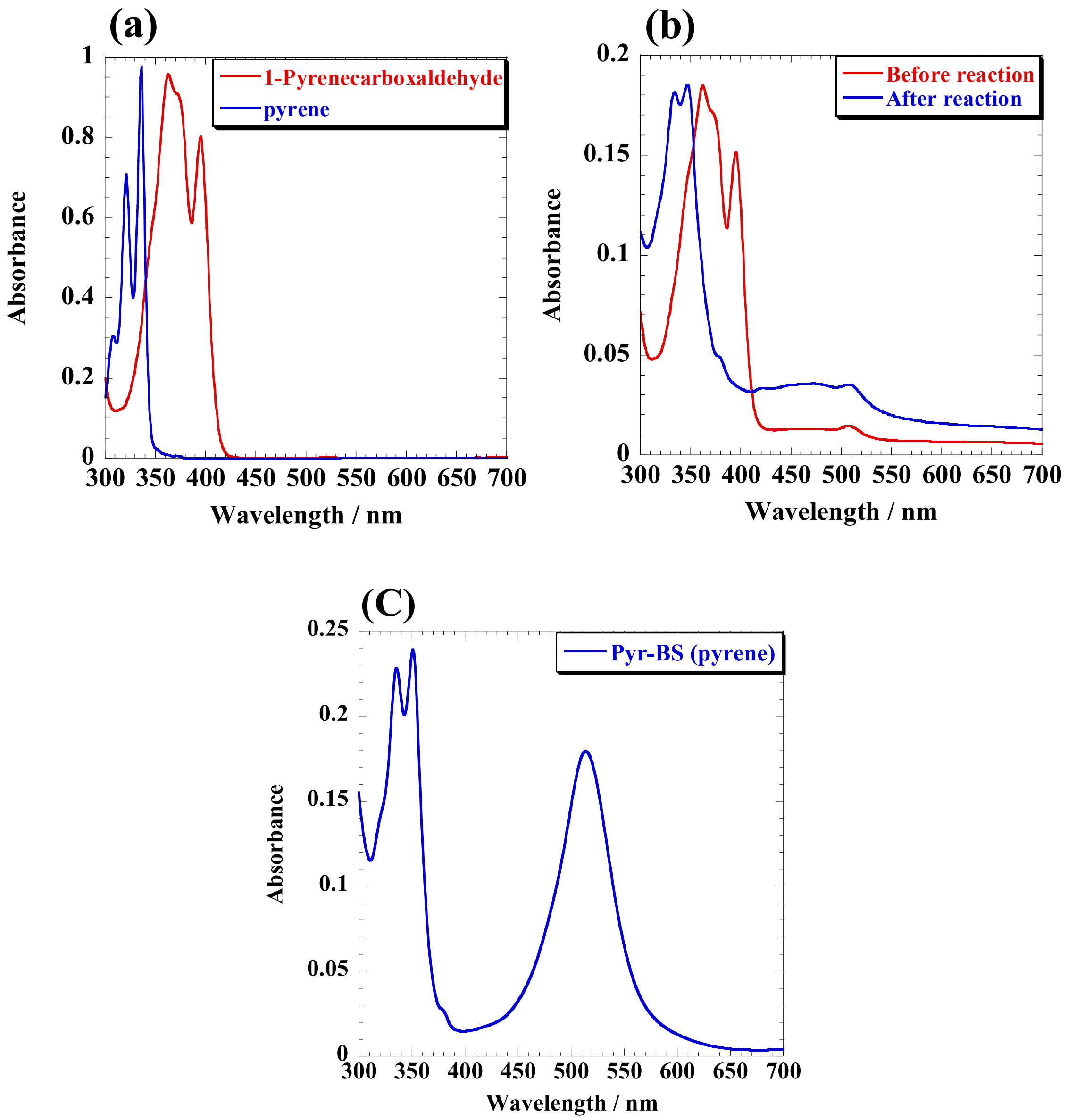

Figure S3. Absorption spectra of (**a**) 1-pyrenecarboxaldehyde and pyrene, (**b**) before and after reaction of **P1** and 1-pyrenecarboxaldehyde dissolved in DMF. Absorption spectrum of (**c**) polymer with pyrene dissolved in phosphate buffer (0.04 g/ L).

(4) Polymers with large numbers of reactive groups

The synthetic routes of the polymers are shown in Scheme S1.

The non-conjugated polymer with TMDA (**P(TMDA)**) was prepared as follows: a solution containing *p*-TS (0.032 g, 0.17 mmol) dissolved in DMF (1.0 mL) was added to a solution of Pyr (0.335 g, **5.0 mmol**), BS (0.937 g, **4.5 mmol**), and TMDA (0.104 g, **0.50 mmol**) dissolved in DMF (5.0 mL) at 10 °C. The resulting solution was stored in the dark at 10 °C for 24 h. The reaction was quenched by the addition of sodium carbonate aqueous solution (5wt%, 3.2 mL). Isopropyl alcohol (80 mL) was added to the reaction mixture. The resulting precipitate, a non-conjugated polymer (**P2**), was purified by serial reprecipitations, twice from DMF/isopropyl alcohol (8 mL/80 mL) and twice from water/isopropyl alcohol (6 mL/80 mL), and was subsequently dissolved in water. The polymer containing TMDA was obtained by freeze-drying (1.196 g). The polymer having pyridine (**P(Pyridine)**, 1.033 g) was prepared in a similar manner.

The π-conjugated polymers containing aldehyde **P(Aldehyde)** and pyridine (**P(Pyridine OX)**) were prepared in a manner similar to that described in Section 2.3.1.

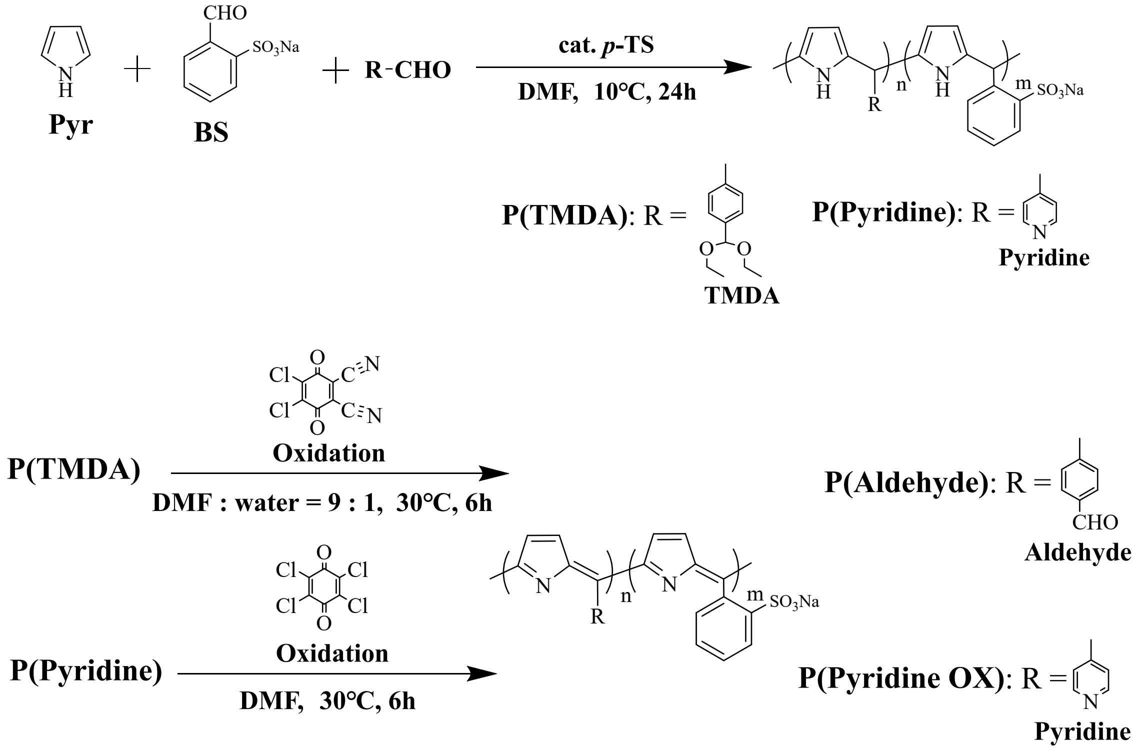

Scheme S1. Synthetic routes of polymers.

Figure S4 shows the chemical structures and ^{1}H-NMR spectra of **P(TMDA) and P(Aldehyde)**. For **P(TMDA)** (**Figure S4a**), the peaks at 9–10 and 5.5 ppm are assigned to the Pyr protons (a and b) and the peaks at 1.3-1.6 ppm are assigned to the protons in the diethyl acetal group (g). After the oxidation reaction, due to the hydrolysis of acetal (TMDA) with the oxidant, the peaks at 1-1.5 ppm (g, the protons in the diethyl acetal group) disappear, and the peaks at 9.6-10.4 ppm (h, the protons in the aldehyde group) appear. The other peaks are consistent with those of the conjugated polymer Pyr(non) (**Figure S4b**).

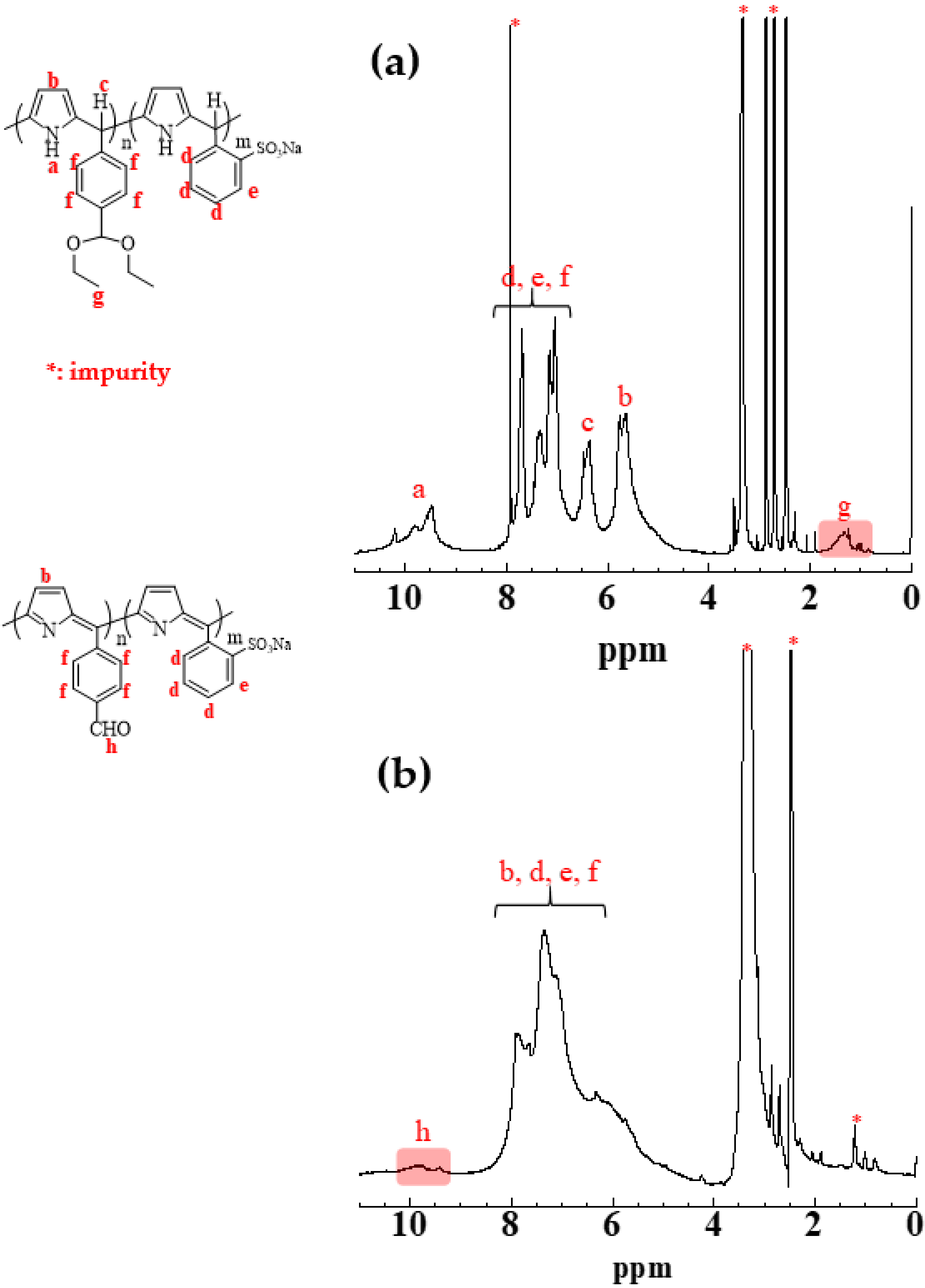

Figure S4. ^{1}H-NMR spectra of (a) P(TMDA) and (b) P(Aldehyde) in DMSO-d6.

Figure S5 shows the chemical structures and ^{1}H-NMR spectra of **P(Pyridine) and P(Pyridine OX)**. For **P(Pyridine)** (**Figure S5a**), the peaks at 9–10 and 5.5 ppm are assigned to the Pyr protons (a and b) and the peaks at 8.3-8.5 ppm are assigned to the protons in the pyridine group (g). After the oxidation reaction, peak broadening is observed from 5

to 9 ppm owing to the oxidation of the conjugated polymers, which causes a toughening of the main chain owing to slow molecular motion. The pyridine protons (f) in the polymer are not assigned, because of spectral broadening and overlapping peaks (**Figure S5b**).

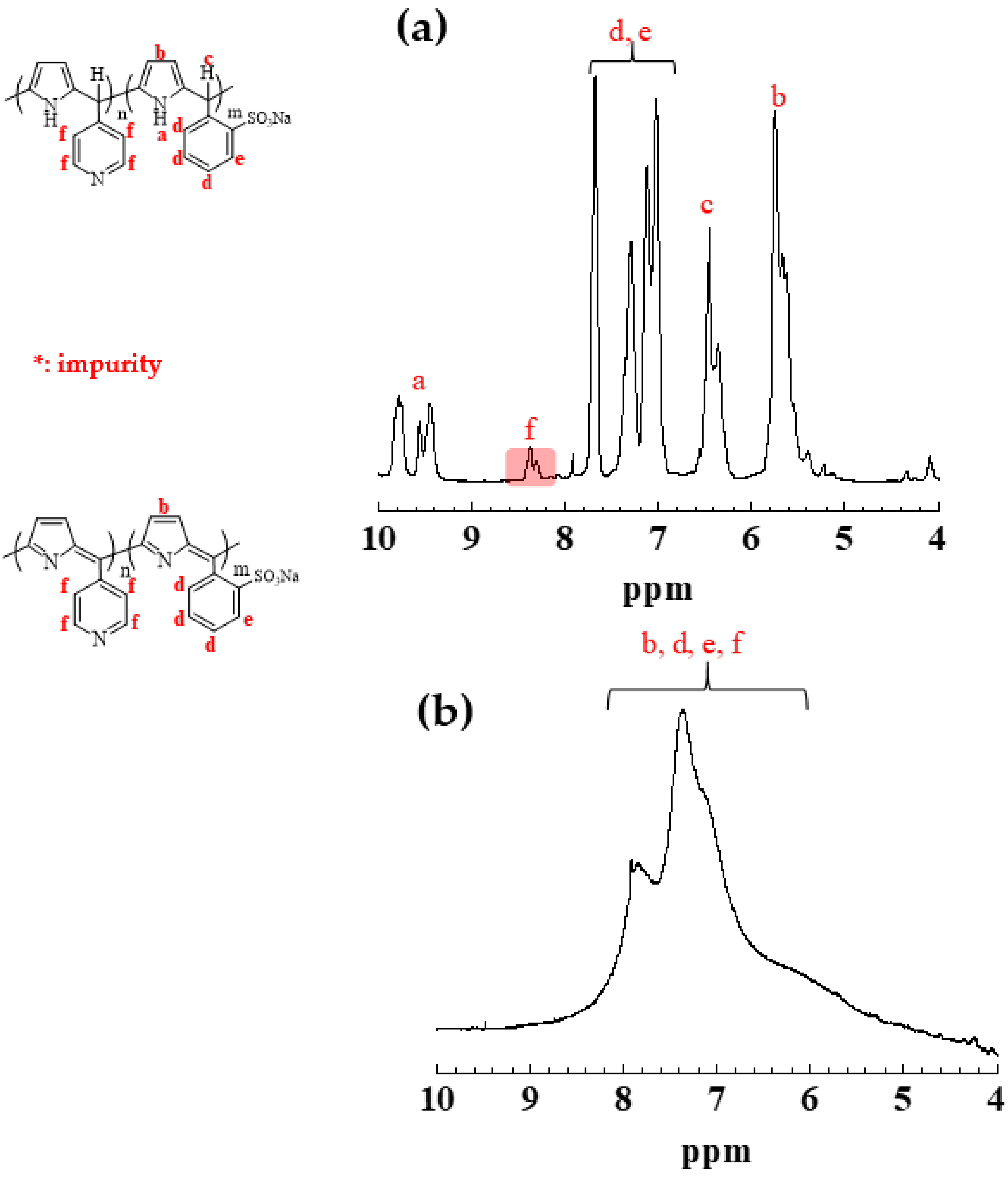

Figure S5. ^{1}H-NMR spectra of (a) P(Pyridine) and (b) P(Pyridine OX) in DMSO-d_6.

(4) UV-Vis-NIR spectra of P2-P5

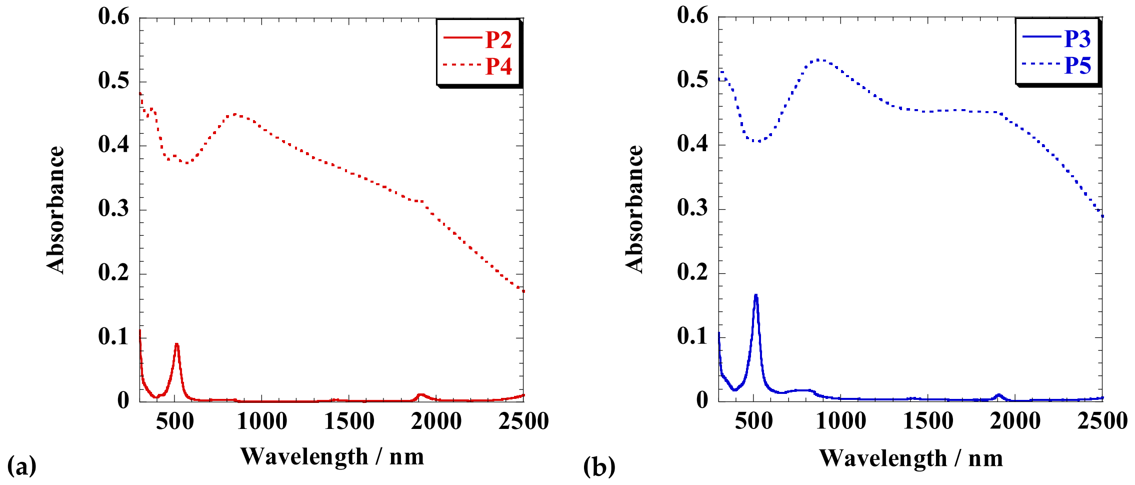

Figure S6. UV-Vis-NIR spectra of polymers (**P2**-**P5**) dissolved in phosphate buffer (4.0 g/ L).

(5) Diffuse reflection spectra of P2, P3, P4, P5, P6 and P7

For the powdered samples, reflection spectra could not be obtained. The diffuse reflectance spectra were measured using an integrating sphere. The vertical axis was converted from reflectance to absorption using the Kubelka–Munk conversion (KM conversion).

Before measuring the diffuse reflection, the polymer powders were dried under vacuum overnight.

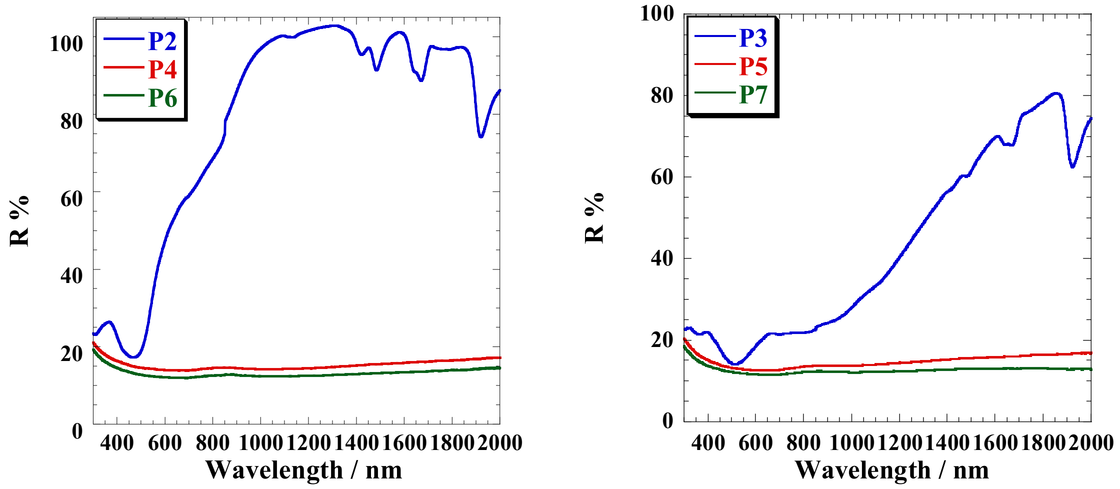

Figure S7. Diffuse reflection spectra of the powdered polymers.

(6) TGA curves of P6 and P7

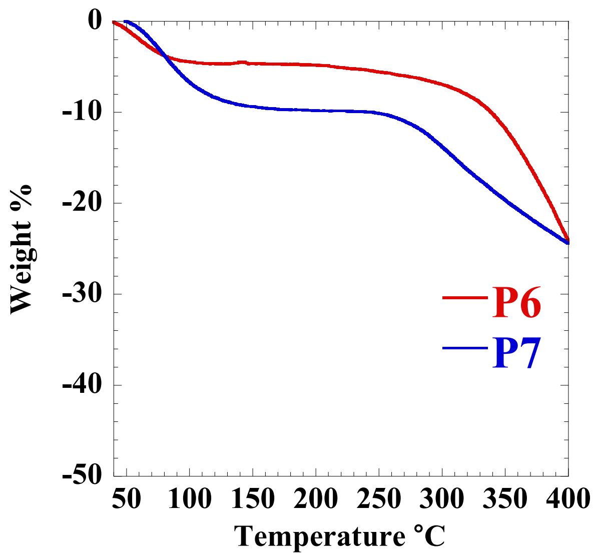

Figure S8. TGA curves of **P6 and P7** with a heating rate of 10 ºC / min under air.

Before measuring thermal gravimetric analysis (TGA), the polymers were dried under vacuum overnight.

(7) DSC curves of P6 and P7

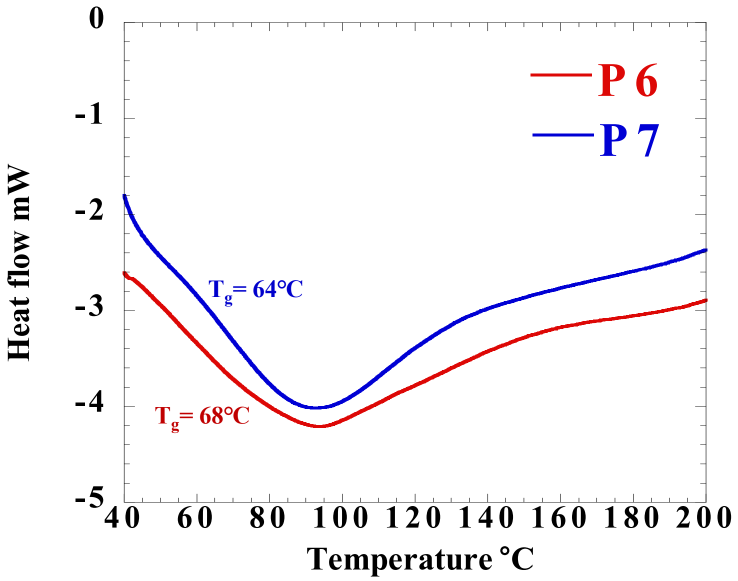

Figure S9. DSC curves of **P6 and P7** with a heating rate of 10 ºC / min under air.

The first feature of the DSC curves indicates that the glass transition temperature (Tg) of the post-crosslinked polymers. The Tg of **P6** and **P7** were 68 and 64°C, respectively.
